# Periodically poled aluminum scandium nitride bulk acoustic wave resonators and filters for communications in the 6G era

**DOI:** 10.1038/s41378-024-00857-4

**Published:** 2025-01-22

**Authors:** Izhar, M. M. A. Fiagbenu, S. Yao, X. Du, P. Musavigharavi, Y. Deng, J. Leathersich, C. Moe, A. Kochhar, E. A. Stach, R. Vetury, R. H. Olsson

**Affiliations:** 1 Department of Electrical and Systems Engineering, University of Pennsylvania, Philadelphia, PA 19104 USA; 2 Department of Materials Science and Engineering, University of Central Florida, Orlando, FL 32816 USA; 3 Akoustis Inc., Huntersville, NC 28078 USA; 4 Department of Materials Science and Engineering, University of Pennsylvania, Philadelphia, PA 19104 USA

**Keywords:** Electrical and electronic engineering, NEMS

## Abstract

Bulk Acoustic Wave (BAW) filters find applications in radio frequency (RF) communication systems for Wi-Fi, 3G, 4G, and 5G networks. In the beyond-5G (potential 6G) era, high-frequency bands (>8 GHz) are expected to require resonators with high-quality factor (*Q*) and electromechanical coupling (${k}_{t}^{2}$) to form filters with low insertion loss and high selectivity. However, both the *Q* and ${k}_{t}^{2}$ of resonator devices formed in traditional uniform polarization piezoelectric films of aluminum nitride (AlN) and aluminum scandium nitride (AlScN) decrease when scaled beyond 8 GHz. In this work, we utilized 4-layer AlScN periodically poled piezoelectric films (P3F) to construct high-frequency (~17–18 GHz) resonators and filters. The resonator performance is studied over a range of device geometries, with the best resonator achieving a ${k}_{t}^{2}$ of 11.8% and a ${Q}_{{\rm {p}}}$ of 236.6 at the parallel resonance frequency (${f}_{{\rm {p}}}$) of 17.9 GHz. These resulting figures-of-merit are (${{{\rm {FoM}}}}_{1}={{k}_{t}^{2}Q}_{{\rm {p}}}$ and ${{{\rm {FoM}}}}_{2}={f}_{{\rm {p}}}{{{\rm {FoM}}}}_{1}{\times }{10}^{-9}$) 27.9 and 500, respectively. These and the ${k}_{t}^{2}$ are significantly higher than previously reported AlN/AlScN-based resonators operating at similar frequencies. Fabricated 3-element and 6-element filters formed from these resonators demonstrated low insertion losses (IL) of 1.86 and 3.25 dB, and −3 dB bandwidths (BW) of 680 MHz (fractional BW of 3.9%) and 590 MHz (fractional BW of 3.3%) at a ~17.4 GHz center frequency. The 3-element and 6-element filters achieved excellent linearity with in-band input third-order intercept point (IIP3) values of +36 and +40 dBm, respectively, which are significantly higher than previously reported acoustic filters operating at similar frequencies.

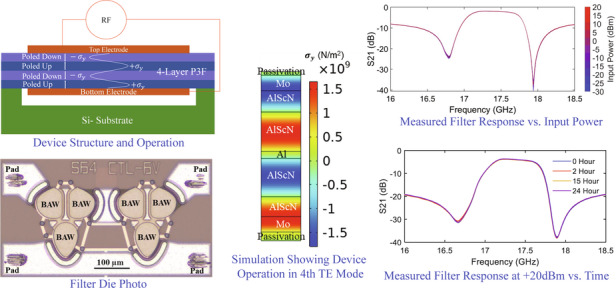

## Introduction

Recent advancements in radio frequency (RF) and mobile communications demand high data rates and small footprints, which require resonators and filters with broader bandwidths and higher frequencies^1,2^. Modern communication standards such as Wi-Fi, 3G, 4G, and 5G have pushed the frequencies beyond traditional bands (<2.6 GHz) to levels as high as 7 GHz, facilitating the accommodation of broader bandwidths^3,4^. In the beyond-5G (potential 6G) era, high-frequency bands (>7 GHz) will be utilized, which are expected to require resonators with high-quality factor (*Q*) and electromechanical coupling (${k}_{t}^{2}$), once RF spectrum use in these bands increases^5^. Acoustic resonators, such as surface acoustic wave (SAW) and bulk acoustic wave (BAW) resonators, have been used predominantly for realizing filters for modern RF communication^3,6^. Scaling SAW resonators to higher frequency requires very thin and delicate interdigitated transducer (IDT) electrode patterns, which suffer from low fabrication yields, poor power handling, and large ohmic losses. For higher frequencies, BAW resonators and filters are preferred due to high ${k}_{t}^{2}$, low insertion loss (IL) and good selectivity. Aluminum nitride (AlN), due to its low dielectric loss tangent, low acoustic damping, and compatibility with fabrication using standard complementary metal-oxide-semiconductor (CMOS) tool sets, has been utilized to implement BAW resonators in the literature^7–10^. Alloying AlN with scandium (Sc) to form aluminum scandium nitride (AlScN) has shown better piezoelectric properties than AlN. For instance, the piezoelectric coefficient, ${d}_{33}$, of AlScN surpasses that of pure AlN by up to five times, showcasing a substantial enhancement in performance^11–13^. The frequency of BAW resonators is strongly dependent on their thickness^14^. Scaling BAW resonators to higher frequencies for beyond-5G applications (>8 GHz) requires a significant reduction in thickness, impacting impedance matching^15^. Though the area of the resonator can be reduced to maintain its input impedance, the area-to-perimeter ratio (*A*/*p*) of the resonator decreases significantly, which leads to degradation in both ${k}_{t}^{2}$ and *Q*^14^. Thus, there is a demand for BAW devices that can operate at elevated frequencies while maintaining equitably thick piezoelectric layers to ensure optimal performance. One strategy to preserve the same layer thickness while achieving a higher operational frequency for BAW devices involves the implementation of periodically poled piezoelectric films (P3F). Barrera et al.^16^ developed acoustic resonators and filters utilizing P3F lithium niobate (LiNbO_3_) achieving a resonator *Q* of 40 and ${k}_{t}^{2}$ of 44% at 19.8 GHz frequency, leading to filter insertion loss (IL) of 2.38 dB and fractional BW of 18.2% at 16.5 GHz. Similarly, Kramer et al.^17^ constructed a P3F LiNbO_3_ acoustic resonator, which obtained ${Q}_{{\rm {s}}}$ of 55 and ${k}_{t}^{2}$ of 40%. However, suspended LiNbO_3_ filters have yet to be commercialized and the initial literature reports show limited linearity, with an in-band, input referred intercept point (IIP3) of +8 dBm in K-band^16^. Utilizing a CMOS compatible 2-layer P3F AlScN, a 13.4 GHz BAW was constructed by Mo et al.^18^, which attained a ${Q}_{{\rm {s}}}$ of 151 and ${k}_{t}^{2}$ of 10.7%. An 18.4 GHz BAW resonator fabricated using P3F AlScN is reported by Vetury et al.^19^ that achieved a ${Q}_{{\rm {p}}}$ of 260 and ${k}_{t}^{2}$ of 7.6%. This was the first time that a P3F AlScN resonator has been constructed in a commercial manufacturing process making it suitable for mass production. Kochhar et al.^20^ reported X-band P3F AlScN resonators and filters achieving a ${Q}_{{\rm {p}}}$ of 789 and ${k}_{t}^{2}$ of 10% at 10.72 GHz frequency leading to filter insertion loss (IL) of 0.7 dB and fractional BW of 4.7% at 10.72 GHz. In our previous work, Izhar et al.^21^ constructed a 20 GHz BAW resonator using a 3-layer P3F AlScN that achieved ${k}_{t}^{2}$ of 8.23% and ${Q}_{{\rm {p}}}$ of 160.

In this work, we fabricate BAW resonators using a 4-layer P3F AlScN, resulting in a >2× increase in the resonator figure-of-merit (FOM) and utilize these resonators to develop filters for RF communication applications in the 6G era. Similar to our prior work^21^, the 4-layer P3F is realized using a single poling step. We report series and shunt BAW resonators required to construct ladder filters, with different sizes (*A*/*p*) and show the trends in *k*_*t*_^2^, *Q*_s_, and *Q*_p_ with device geometry. The resonators achieved a maximum ${Q}_{{\rm {p}}}$ of 236.6 at a parallel resonance frequency (${f}_{\rm {{p}}}$) of 17.9 GHz and a maximum ${k}_{t}^{2}$ of 11.8%, which resulted in higher figures-of-merits (${{{\rm {FoM}}}}_{1}=27.9$ and ${{{\rm {FoM}}}}_{2}=500$) compared to state-of-the-art AlN and AlScN K and Ku band resonators. Filters using different topologies (3-element and 6-elements) were constructed from the P3F AlScN BAW resonators achieving low IL of 1.86 and 3.25 dB, and −3 dB bandwidths (BW) of 680 MHz (fractional BW of 3.9%) and 590 MHz (fractional BW of 3.3%) at a ~17.4 GHz center frequency. The filters demonstrated power handling > + 20 dBm without any measurable compression and showed no change in response after a 24 h in-band power soak at +20 dBm. The 6-element filter demonstrated a test setup limited in-band, input referred third order intercept point (IIP3) > + 40 dBm. Both the filter power handling and linearity are orders of magnitude higher than recent acoustic filter demonstrations at similar frequencies^6,16^.

### Design and fabrication

**Fig. 1:**
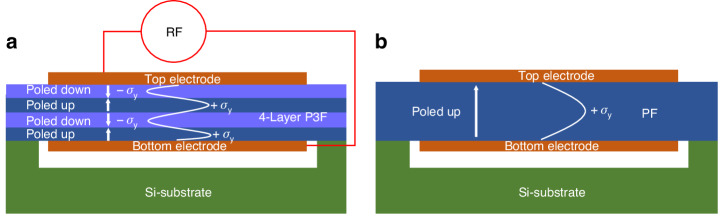
Structure and working principle. **a** P3F AlScN resonator operating in the 4th thickness extensional mode (TE4) with operating frequency 4 times higher than **b** similar unpoled resonator operating at the fundamental thickness extensional mode (TE1)

The structure and working mechanism of the P3F AlScN BAW resonator is illustrated in Fig. 1. The resonator is comprised of a 4-layer AlScN P3F, as grown and electrically poled, sandwiched between top and bottom molybdenum (Mo) electrodes. Under electrical excitation, the device experiences contraction and expansion, due to different polarities of the AlScN, inducing tensile (+${\sigma }_{y}$) and compressive (−${\sigma }_{y}$) stresses in the fourth thickness extensional (TE4) mode. This process produces acoustic waves within the device which resonate at a frequency of approximately

1 $${f}_{{\rm {P3F}}}=\frac{v}{\lambda }=\frac{4v}{2t}$$

where $\lambda$, $v$ and $t$, respectively denote the acoustic wavelength, acoustic velocity, and thickness of the resonator. The P3F AlScN enabled the resonator to operate at four times higher frequency compared to a similar unpoled resonator

2 $${f}_{{{\rm {PF}}}}=\frac{v}{\lambda }=\frac{v}{2t}\,$$

operating in the fundamental thickness extension mode (TE1) as depicted in Fig. 1b. The P3F BAW resonators and filters are constructed from AlScN because the electromechanical coupling^22^

3 $${k}_{t}^{2}=\frac{\,{d}_{33}^{2}{E}_{3}}{\varepsilon }$$

for a BAW resonator (neglecting the metal electrodes) depends on piezoelectric coefficient (${d}_{33}$), modulus of elasticity (${E}_{3}$) and dielectric constant ($\varepsilon$). The ${d}_{33}$ of ${{\rm{Al}}}_{0.64}{{\rm{Sc}}}_{0.36}{\rm{N}}$ (~22.9 pm/V) is much higher than other piezoelectric materials such as AlN (3.9 pm/V) and ZnO (5.9 pm/V), therefore it enables the BAW resonators to achieve high ${k}_{t}^{2}$ ^23,24^.

**Fig. 2:**
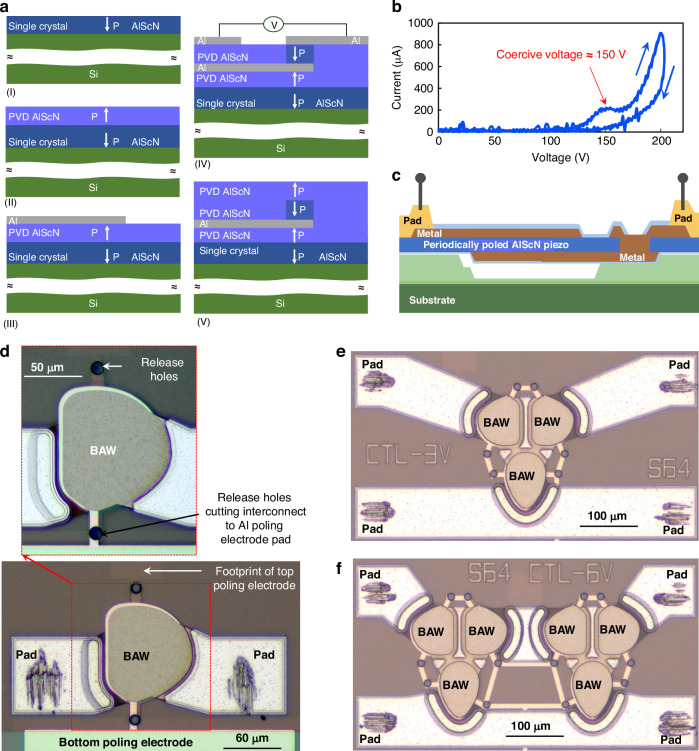
Resonator and filter fabrication and poling waveform. **a** The fabrication process for P3F AlScN involves: (I) deposition of an$\,{{\rm{Al}}}_{0.8}{{\rm{Sc}}}_{0.2}{\rm{N}}$ (layer 1) in a M-polar orientation, (II) PVD deposition of$\,{{\rm{Al}}}_{0.64}{{\rm{Sc}}}_{0.36}{\rm{N}}$ (layer 2) in a N-polar orientation, (III) sputtering of Al, serving as the bottom electrode for poling, (IV) PVD deposition of$\,{{\rm{Al}}}_{0.64}{{\rm{Sc}}}_{0.36}{\rm{N}}$ (layer 3), Al top electrode for poling, and electrical poling of deposited$\,{{\rm{Al}}}_{0.64}{{\rm{Sc}}}_{0.36}{\rm{N}}$ (layer 3), and finally, (V) stripping off the top Al poling electrode and PVD deposition of$\,{{\rm{Al}}}_{0.64}{{\rm{Sc}}}_{0.36}{\rm{N}}$ (layer 4). **b** The current vs. voltage response recorded during the electrical poling of the AlScN (layer 3) in step IV, showing the transition of AlScN (layer 3) from N-polar to M-polar. This waveform underlines the critical switching coercive voltage point, which registers at ~150 V. **c** Cross-sectional view of the BAW resonator realized in the XBAW process. **d** Optical micrograph of a BAW resonator showing the device pads and poling electrodes, and optical micro image of the fabricated **e** 3-element and **f** 6-element filters

The resonators were fabricated by (1) the formation of as grown and electrically poled 4-layer AlScN P3F on a 6-inch silicon wafer, and (2) then subsequently transforming the P3F into BAW resonators through the utilization of the commercial XBAW^TM^ process. The detailed process flow for the construction of the P3F AlScN 4-layer stack is described in Fig. 2a. The P3F formation initiated with the deposition of 124 nm of$\,{{\rm{Al}}}_{0.8}{{\rm{Sc}}}_{0.2}{\rm{N}}$ (layer1) in a Metal-polar (M-polar) orientation (poled down), utilizing the technique reported by Vetury et al.^19^ and Moe et al.^25^ (step I). Next, a physical vapor deposition (PVD) co-sputtering process was employed to deposit 230 nm of$\,{{\rm{Al}}}_{0.64}{{\rm{Sc}}}_{0.36}{\rm{N}}$ (layer 2) in a Nitrogen-polar (N-polar) orientation (step II). Following this, a 36 nm-thick aluminum (Al) was sputtered in the same process chamber without a vacuum break to avoid oxide formation on the AlScN (step II). This was followed by patterning of the Al layer to form the bottom electrode for poling purposes. Another layer of 223 nm thick ${{\rm{Al}}}_{0.64}{{\rm{Sc}}}_{0.36}{\rm{N}}$ (layer 3) in an N-polar orientation (poled up) was deposited via the PVD co-sputtering technique during the subsequent step (step IV). Following this, a 40 nm-thick Al layer was sputtered using the same PVD system, and then patterned using wet etching chemistry to form the top electrode for electrical poling of ${{\rm{Al}}}_{0.64}{{\rm{Sc}}}_{0.36}{\rm{N}}$ (layer 3). Using the Al electrodes, the top ${{\rm{Al}}}_{0.64}{{\rm{Sc}}}_{0.36}{\rm{N}}$ (layer 3) was then electrically poled, transitioning from N-polar (poled up) to M-polar (poled down). Figure 2b presents the current vs. applied voltage curve, demonstrating the switching of the top AlScN layer from N-polar orientation to M-polar orientation. The coercive voltage of ~150 V was identified during ferroelectric switching by the abrupt increase in current upon ferroelectric switching of the layer. The poling was achieved at the filter level where 3 and 6 BAW resonators were poled in a single poling step. Additional details on the electrical poling are included in the supplementary data. The top Al was removed in the subsequent step (V). The bottom Al poling electrode cannot be removed after poling, and its presence can potentially degrade the ${k}_{t}^{2}$ and *Q* of the resonators. To minimize its impact on device performance the acoustic layer stack is engineered to minimize the stress field in the Al poling electrode by minimizing its thickness and placing it at the interface between two periodically poled regions where a null in the stress field is observed. If the remaining Al poling layer can be limited to be within the active P3F BAW device region, there is no added parasitic capacitance from the Al layer, and it does not distort the desired electric field or the device operation from that schematically depicted in Fig. 1a. The key item is to limit any remaining Al poling layer outside the active BAW area. This can add extra parasitic capacitance that can degrade the *k*_*t*_^2^. To minimize parasitic capacitance arising from the interconnects to the Al poling layer, the remaining lines are cut in close proximity to the BAW resonators during etching of the piezoelectric layer as part of the XBAW^TM^ process, as shown in Fig. 2d–f. Furthermore, any overlap between the remaining poling Al layer and the top or bottom BAW electrodes outside the active P3F BAW device region can also add parasitic capacitance and degrade the *k*_*t*_^2^. Thus, maintaining precise alignment between the embedded poling metal and the top/bottom BAW electrodes during fabrication is critical for achieving high *k*_*t*_^2^. Finally, a 115 nm thick ${{\rm{Al}}}_{0.64}{{\rm{Sc}}}_{0.36}{\rm{N}}$ (layer 4) was deposited via co-sputtering to realize the 4-layer AlScN P3F. The surface of the manufactured AlScN P3F underwent analysis using an atomic force microscope (AFM), revealing a surface roughness of <2 nm.

The 6-inch wafers with the 4-layer AlScN P3F were sent to Akoustis Inc., where a commercial XBAW^TM^ process was used to construct BAW resonators and filters from the P3F. The cross-sectional depiction of a typical BAW resonator, accomplished through the XBAW^TM^ process, is illustrated in Fig. 2c. Detailed information on the XBAW^TM^ process can be found in the pertinent literature^19,26^ The critical dimensions of the fabricated resonators and filters, which enabled the devices to operate TE4, are provided in the supplementary data.

## Results and discussion

A vector network analyzer, VNA (Keysight Technologies E8361A PNA) was used to characterize the P3F resonators and filters (see Supplementary Information). A signal-ground (SG) probe (ACP-40 SG 200, FormFactor Inc., USA) was used to connect the resonator to the VNA. One port measurements were taken with an input power of −10 dBm, a port impedance of 50 Ω, and a frequency range of 3–25 GHz. Calibration to the probe tips was accomplished using the short-open-load (SOL) technique^27^.

The data extracted using the VNA contains the on-wafer interconnects to the GS probe in addition to the intrinsic resonator response. De-embedding was performed to obtain the response of the intrinsic resonator from the measured reflection factor (Γ_M_) using equivalent open (Γ_M,open_) and short (Γ_M,short_) structures produced on the same Si-wafer^28^.

**Fig. 3:**
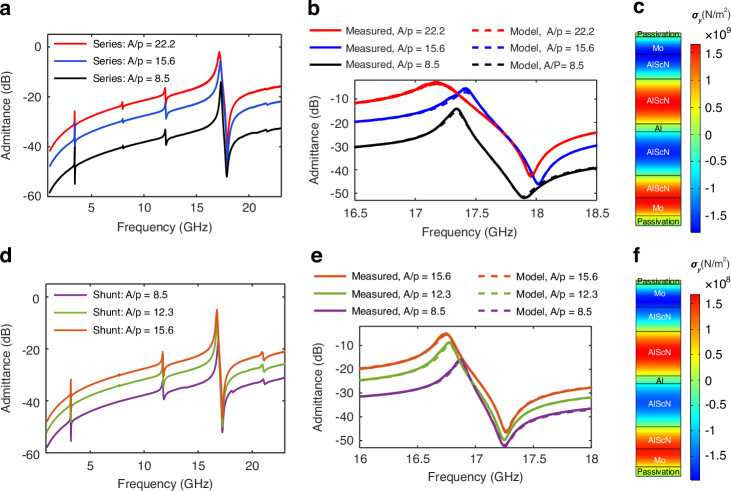
Measured results and simulated mode shapes of resonators. Admittance responses of **a** and **b** series and **d** and **e** shunt resonators showing their operation at the fourth thickness extensional mode (TE4). The stress distribution of the **c** series and **f** shunt resonators confirms the TE4 mode of vibration for the devices

The admittance responses of the resonators after de-embedding are shown in Fig. 3a. It is observed from the figure that the devices exhibit distinct vibration modes corresponding to the first (TE1), second (TE2), third (TE3) and fourth (TE4) thickness extension (TE) modes at approximately 3.5, 8, 12, and 18 GHz frequencies, respectively. The figure illustrates that the devices show a dominant response at TE4 (~18 GHz). This observation also confirms the successful poling of the devices, as depicted in Fig. 1, where the P3F devices were specifically engineered to operate optimally in the TE4 mode (see supplementary data showing measurements of an identical unpoled resonator where TE1 is the dominant mode). To further clarify these findings, simulations were conducted using COMSOL Multiphysics to analyze the vibration modes of the devices. As shown in Fig. 3c, the stress distributions of the acoustic standing wave at ~18 GHz frequency in the devices indicate their operation in the TE4 mode. The stress distributions at ~3.5, 8 and 12 GHz frequencies, provided in the supplementary data, confirm the TE1, TE2, and TE3 modes of operation of the device do not match the periodically poled AlScN, and thus those modes have reduced ${k}_{t}^{2}$.

Furthermore, shunt resonators with slightly thicker metal electrodes were exploited towards the implementation of ladder filters, with measured responses shown in Fig. 3d, e. These resonators also operated in the TE4 mode, which is clear from the mode shapes shown in Fig. 3f. However, due to the thick top metal electrodes, these resonators have slightly lower resonant frequencies (${f}_{{\rm {p}}}$ ~ 17.25 GHz) than the series resonators (${f}_{{\rm {p}}}$ ~ 18 GHz), which are utilized to realize broadband filters.

A modified Butterworth Vandyke (mBVD) model, as presented in Table 1, consisting of a series resistance (${R}_{{\rm {s}}}$), a motional resistance (${R}_{{\rm {m}}}$), capacitance (${C}_{{\rm {m}}}$) and inductance (${L}_{{\rm {m}}}$), and a shunt capacitance (${C}_{0}$) and resistance (${R}_{o}$), is used to fit the measurements results of both series and shunt resonators^29^. The measurement results in terms of admittance response of both series and shunt resonators are in close agreement with the mBVD model as shown in Fig. 3b and e, respectively. All the devices showed very clean responses in their region of operation, and no unwanted spurs were observed. Devices with higher *A*/*p* ratio exhibited higher admittance which is due to their higher values of shunt capacitance compared to devices with smaller *A*/*p* ratio.

The Bode *Q*^30,31^

4 $$Q\left(\omega \right)=\omega \frac{{{d}}\varphi }{{{d}}\omega }\frac{\left|{S}_{11}\right|}{1-{\left|{S}_{11}\right|}^{2}}$$

of the devices were calculated in terms of *S*-parameter (${S}_{11}$), phase ($\varphi$) and frequency ($\omega$). The Bode *Q* of both the measurement data and mBVD model fit very closely as provided in supplementary data.

**Fig. 4:**
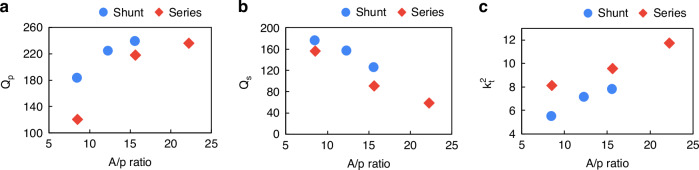
Performance parameters of the resonators as a function of their dimension. **a** ${Q}_{{\rm {p}}}$ of both series and shunt resonators increases whereas **b** ${Q}_{{\rm {s}}}$ decreases with an increase in the *A*/*p* of the device. **c** ${k}_{t}^{2}$ of both series and shunt resonators increase with increasing device A/*p*

The performance parameters for all the devices in terms of parallel quality factor (${Q}_{{\rm {p}}}$), series quality factor (${Q}_{{\rm {s}}}$), and electromechanical coupling (${k}_{t}^{2}$)

5 $${k}_{t}^{2}=\frac{{\pi }^{2}}{8}\left[\frac{{f}_{{\rm {p}}}^{2}-{f}_{{\rm {s}}}^{2}}{{f}_{{\rm {s}}}^{2}}\right]$$

are summarized in Fig. 4a–c, respectively. It is clear from Fig. 4a that the ${Q}_{{\rm {p}}}$ increases with an increase in the *A*/*p* ratio for the devices. This is because, with an increase in the *A*/*p* ratio, the acoustic loss to the anchor boundaries also decreases which helps improve the ${Q}_{{\rm {p}}}$^14^. A maximum ${Q}_{{\rm {p}}}$ of 236. 6 is achieved by the series resonator with an *A*/*p* ratio of 22.2. While the ${Q}_{{\rm {p}}}$ increases, the ${Q}_{\rm {{s}}}$ of both series and shunt resonators decreases with increasing *A*/*p* ratio as shown in Fig. 4b. This is because, while ${Q}_{{\rm {p}}}$ is dominated by the acoustic loss, the ${Q}_{{\rm {s}}}$ is dominated by the ohmic loss. Since the resonators with higher *A*/*p* ratio have smaller motional resistances, they are impacted more by the series resistance hence lowering their ${Q}_{{\rm {s}}}$^14^. The *Q* of the shunt resonators is slightly higher than the series resonators for the same *A*/*p* ratio. This is because of the mass loading effect of the thick Mo top electrodes, which helped the shunt resonators achieve higher ${L}_{m}$ compared to the series resonators with thin electrodes and the reduced ${R}_{{\rm {s}}}$ from the thicker metal (see Table 1), thus improving their ${Q}_{{\rm {p}}}$ and ${Q}_{{\rm {s}}}$. The ${k}_{t}^{2}$ of the resonators, as shown in Fig. 4c, increases with an increase in the *A*/*p* ratio. This is because the center part of the resonator (proportional to *A*) is free to move, while the periphery of the resonator (proportional to *p*) is attached to the substrate and does not vibrate. Therefore, with an increase in *A*/*p* ratio more electrical energy is converted into acoustic energy within the resonator thus improving ${k}_{t}^{2}$ ^14^. A maximum ${k}_{t}^{2}$ of 11.8% is achieved by the series resonator with an *A*/*p* ratio of 22.2, which resulted in a figure-of-merit (${{{\rm {FoM}}}}_{1}$ = ${k}_{t}^{2}$. ${Q}_{{\rm {p}}}$) of 27.9 for the same resonator.

**Table 1:** The mBVD model parameters and values fit to experimental results for the series and shunt resonators

mBVD model	Resonator	*A*/*p* ratio	*R*_s_ (Ω)	*C*_o_ (pF)	*R*_m_ (Ω)	*L*_m_ (nH)	*C*_m_ (fF)	*R*_o_ (Ω)
	Series	22.2	1.142	1.2552	0.385	0.755	113.407	0.015
		15.5	1.195	0.6042	1.28	2.013	41.436	0.022
		8.5	1.132	0.1761	4.75	7.656	10.995	2.157
	Shunt	15.5	0.813	0.6137	1.1	2.384	37.863	0.052
		12.3	0.971	0.3576	2.121	4.476	20.097	0.097
		8.5	0.905	0.184	5.89	10.839	8.205	0.217

**Fig. 5:**
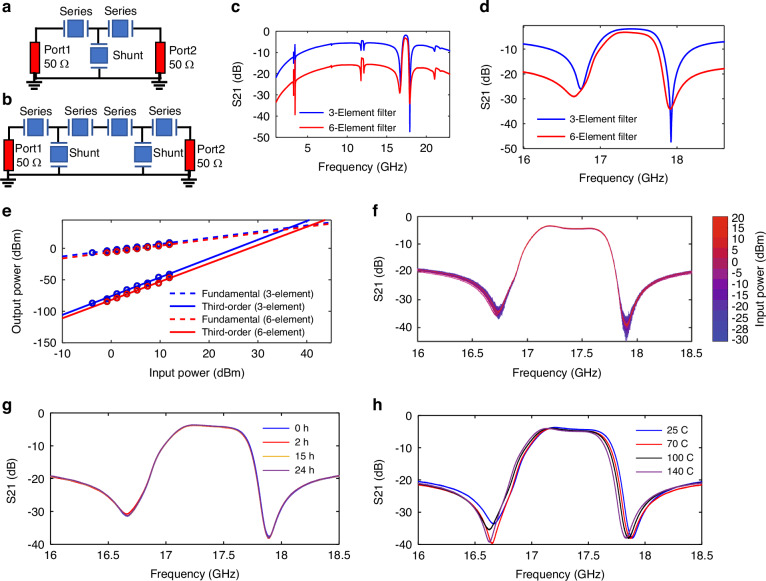
Measured results of 3 and 6-element P3F filters. Schematic of **a** three and **b** six elements filters. Broadband **c** and **d** narrow band response of the 3 and 6 element filters showing IL of 1.86 and 3.25, and −3 dB bandwidths (BW) of 680 MHz (fractional BW of 3.9%) and 590 MHz (fractional BW of 3.3%), respectively. Input and output power of the filters showing **e** In-band IIP3 values of +36 and +40 dBm for the three and six-element filters, **f** Response of 6-element filters at different input power levels showing linear operation up to +20 dBm, which is the limit of our test equipment, **g** Response of the 6-element filter recorded after 0, 2, 15, and 24 h power soak with a +20 dBm input tone applied in the filter passband, and **h** change in frequency of the 6-element filter with increase in ambient temperature showing a minor impact on the filter

High-frequency bandpass filters using different topologies (such as 3-element and 6-element) were constructed from the P3F AlScN BAW resonators as shown in Fig. 5a and b, respectively. A relatively simple design for the filter is achieved by the arrangement of series-shunt-series resonators as shown in Fig. 5a. Though this design achieves low IL, it offers low out-of-band rejection. Therefore, another 6-element filter is demonstrated by connecting two 3-element filters in series, as shown in Fig. 5b. This design is intended to offer better rejection which is useful for blocking unwanted frequencies and attenuating interference.

Two Signal-Ground (SG) probes (ACP-40 SG 200, FormFactor Inc., USA) were used to connect the filters to the VNA (see Supplementary Information). Two port measurements were taken with an input power of −10 dBm, a port impedance of 50 Ω, and a frequency range of 3–25 GHz. Calibration to the probe tips was achieved using the short-open-load-thru (SOLT) technique^27^. The 3-element filter achieved a low IL of 1.86 dB and −3 dB bandwidth (BW) of 680 MHz (fractional BW of 3.9%) at a ~17.4 GHz center frequency, which is expectedly better than the IL (3.25 dB) and BW (590 MHz, fractional BW of 3.3%) of the 6-element filters. Whereas the out-of-band rejection achieved by 6-element filters is 16.6 dB, which is much better than the out-of-band rejection (~7 dB) achieved by the 3-element filters. The power handling and linearity of the filters were also analyzed, revealing in-band IIP3 values of +36 and +40 dBm for the 3- and 6-element filters, respectively, as depicted in Fig. 5e. The measured values are limited by the experimental setup as an IIP3 of similar value is obtained for a standard thru structure on a calibration substrate (see Supplementary Information)^32^. The response of the 3- and 6-element filters at different input power levels is shown in Fig. 5f and in Supplementary Information. Both filters show linear operation up to +20 dBm, which is the limit of our test equipment and much higher than other state-of-the-art acoustic filters operating at similar frequency^6,16^. Moreover, to test the stability of the 6-element filter, it was operated continuously at +20 dBm in-band input power for 1 day. The measured response of the filter after different power soak durations, as shown in Fig. 5g, clearly shows that the device behavior does alter over the power soak, which confirms the long-term stability of the device. To analyze the response of the filter to temperature, it was tested from room temperature to 140 °C. As expected, the operating frequency of the filter slightly decreases with increasing temperature due to the reduced acoustic velocity of the AlScN, Al, and Mo layers at elevated temperatures. Similar behavior is seen in traditional BAW resonators and filters at lower frequencies^33^. The P3F AlScN-based filter showed a temperature coefficient of frequency (TCF) of −9.63 ppm/K. To improve the thermal stability a temperature compensation layer (usually SiO_2_) can be integrated into the layer stack^33^, but this will reduce the *k*_*t*_^2^. For wideband filters with relatively low TCF, such as the filters reported here shown in Fig. 5h, temperature compensation is often not required as the total frequency shift is small compared to the bandwidth.

The performance of the P3F resonators and filters reported in this work are compared with the existing state-of-the-art in Table 2. The *k*_*t*_^2^ and FoM obtained of 11.8% and 27.9 are much higher than that previously reported for AlN/AlScN-based resonators operating in K and Ku bands, owing to the increased number of P3F layers and the use of 36% Sc alloyed AlScN. The inclusion of additional P3F layers allows for an increase in the device’s *A*/*p* ratio while still maintaining an impedance near 50 Ω required for filter applications. A larger *A*/*p* ratio increases the stored mechanical energy in the resonator when compared to the acoustic losses to the substrate and the electrical stored energy in the resonator periphery, thereby facilitating the attainment of higher *Q*_p_ and *k*_*t*_^2^ as observed in Fig. 4. Moreover, the *Q* of the AlScN BAW devices benefits from resonators which have thick metal electrodes with low electrical resistance, but that comes with the cost of reduced *k*_*t*_^2^ as more mechanical energy is stored in the non-piezoelectric metal regions. Increasing the number of P3F layers in a resonator while maintaining a fixed thickness for the metal electrodes results in more of the mechanical strain energy being stored in the piezoelectric layers, which contributes to the electromechanical coupling. Thus, higher numbers of P3F layers result in larger *k*_*t*_^2^*Q* products resulting in improved resonator and filter small signal performance. While the FOM of LiNbO_3_ resonators is higher due to the larger *k*_*t*_^2^, the higher *Q* of AlScN P3F resonator technology makes it more appropriate for realizing low-loss filters with narrower passbands <8%. Also, LiNbO_3_ resonators are not fully compatible with CMOS technology.

**Table 2:** Comparison of the developed resonators and filters with the existing state-of-the-art

Reference	P3F material	Fully CMOS compatible	Resonator performance							Filter			
			Freq. (GHz)	*A*/*p* ratio	Type	*Q*_s_	*Q*_p_	${k}_{t}^{2}$ (%)	${{{\rm {FoM}}}}_{1}$	IL (dB)	BW (%)	Rejection (dB)	IIP3 (dBm)
Lu et al.^35^	LiNbO_3_	Non-CMOS	19.5	29	–	NR	445	1.26	5.6	7.86	0.21	–	–
Kramer et al.^17^	LiNbO_3_	Non-CMOS	19.2	–	–	55	–	40	22	–	–	–	–
Barrera et al.^16^	LiNbO_3_	Non-CMOS	19.8	–	Shunt	40	–	44	17.6	2.38	18.2	13	8
Barrera et al.^36^	LiNbO_3_	Non-CMOS	38.2	–	Shunt	13	–	30	38.7	5.63	17.6	10.2	–
Mo et al.^18^	AlScN	CMOS	13.9	–	Series	151	NR	10.7	16.2	–	–	–	–
Kochhar et al.^20^	AlScN	CMOS	10.72	–	Shunt	347	789	10	79	0.7	4.7	6	–
Izhar et el.^21^	AlScN	CMOS	20	16	Series	92	160	8.23	13.2	–	–	–	–
Vetury et al.^19^	AlScN	CMOS	18.4	–	Series	180	260	7.6	20	–	–	–	–
Schaffer et al.^37^	AlN	CMOS	55.7	2.7	Series	93	89	2.2	2.1	–	–	–	–
Cho et al.^38^	AlScN	CMOS	21	2.3	Series	62	32	6.4	2.1	–	–	–	–
	AlScN	CMOS	55.4	2.3	Series	30	27	3.8	1.1	–	–	–	–
This work	AlScN	CMOS	17.4	22.2	Series	58.4	236.6	11.8	27.9	3.25	3.4	16.6	>40 dBm

The series and shunt resonators developed in this work were utilized to implement ladder filters. The high-frequency filters (~18 GHz) developed in this work showed much higher linearity, power handling, and out-of-band rejection when compared to the existing state-of-the-art acoustic filters at similar frequencies. When increasing the number of P3F layers for a fixed resonator impedance, which is often dictated by the filter requirements and topology, the volume of the resonator increases proportional to the square of the number of P3F layers. Thus, the resonator power density at a given incident RF power decreases proportional to the square of the number of P3F layers and the power handling and linearity are improved.

This work reports 4-layer P3F by combining the as-grown 2-layer P3F reported by Vetury et al.^19^, with the electrically poled P3F reported by Izhar et al.^21^. The use of ${{\rm{Al}}}_{0.64}{{\rm{Sc}}}_{0.36}{\rm{N}}$ for electrical poling, as opposed to ${{\rm{Al}}}_{0.68}{{\rm{Sc}}}_{0.32}{\rm{N}}$ in the prior report^21^, resulted in much higher yield for the electrical poling, facilitating the study of numerous resonator geometries and filters and understanding of the trends in *Q*_s_, *Q*_p_, and *k*_*t*_^2^ with *A*/*p*. While this work represents a significant advance for AlScN resonators and filters operating in Ku-band, there are several remaining challenges for integrating these filters into communications systems. Currently, the electrical poling is performed at the filter level and is not automated, which is time-consuming and represents a barrier to mass production. Future studies will determine the largest area that can be poled in a single poling step with a high yield and will automate the electrical poling process. While the FOM achieved of 27.9 is a large increase compared to prior AlScN works at similar frequencies, a higher FOM is needed to reduce filter insertion loss while increasing the out-of-band rejection. The trend in *k*_*t*_^2^ with *A*/*p* in Fig. 4 clearly indicates that further increases in *A*/*p*, which would be facilitated by additional P3F layers, would result in higher *k*_*t*_^2^ and, thus higher FOM. We are far from the *k*_*t*_^2^ limit for the ${{\rm{Al}}}_{0.64}{{\rm{Sc}}}_{0.36}{\rm{N}}$ material. For example, a prior study of BAW resonators using a traditional 380 nm-thick ${{\rm{Al}}}_{0.68}{{\rm{Sc}}}_{0.32}{\rm{N}}$ of uniform polarization yielded a *k*_*t*_^2^ of 23.1% at 4.8 GHz^34^. As discussed above, an increased number of P3F layers for a given A/p ratio also results in higher *Q*_s_. Thus, a key to additional FOM improvement is to further increase the number of P3F layers beyond 4. An ideal solution from a manufacturability perspective would be innovative methods to grow highly Sc alloyed M-polar AlScN directly on N-polar AlScN. Another approach would be to automate the poling process and to increase the area that can be electrically poled with high yield to make multiple P3F layers realized via electrical poling more practical.

## Conclusion

In this study, we employed P3F AlScN to fabricate resonators and filters operating at high frequencies (~18 GHz). The resonator with the highest *A*/*p* ratio achieved a ${Q}_{{\rm {p}}}\,$ value of 236.6 at a ${f}_{\rm {{p}}}$ of 17.9 GHz and a ${k}_{t}^{2}$ value of 11.8%. This led to figures of merit (${{{\rm {FoM}}}}_{1}={{k}_{t}^{2}Q}_{{\rm {p}}}$ and ${{{\rm {FoM}}}}_{2}={f}_{{\rm {p}}}{{{\rm {FoM}}}}_{1}{\times }{10}^{-9}\,$) of 27.9 and 500 for the resonator, respectively. These figures of merit surpass those of existing state-of-the-art resonators realized in AlN and AlScN materials operating at similar or higher frequencies. The 3-element and 6-element filters exhibited low IL of 1.86 and 3.25 dB, along with −3 dB bandwidths (BW) of 680 MHz (3.9% fractional BW) and 590 MHz (3.3% fractional BW) at a center frequency of ~17.4 GHz. Both filters demonstrated excellent linearity, with in-band IIP3 values of > + 40 dBm. It is concluded from the experimental results that P3F AlScN resonator and filter technology holds promise for advancing RF communications in the beyond-5G era.

## Methods and materials

### Microfabrication of periodically poled piezoelectric film (P3F)

The periodically poled piezoelectric film (P3F) was produced on 6-inch Silicon wafers at the University of Pennsylvania. The wafer was first sent to the AKTS foundry for printing alignment features. This was done to align the in-house processing steps to those performed in the AKTS foundry. The first layer deposition of 124 nm of$\,{{\rm{Al}}}_{0.8}{{\rm{Sc}}}_{0.2}{\rm{N}}$ (layer1) in an M-polar orientation (poled down) was also performed in the AKTS foundry. The wafers received from the foundry were processed in a PVD system to deposit subsequent layers of AlScN. In the PVD system, the targets underwent conditioning to ensure the deposition of high-quality AlScN. Utilizing separate 4-inch diameter Al and Sc targets, along with nitrogen gas, facilitated the deposition process. Prior to depositing each AlScN layer, the process chamber underwent pumping to attain a base pressure of ~9 × 10^–8^ mbar, and the substrate temperature was elevated to 350 °C. To attain the desired composition of 36% Sc in the deposited film, the respective cathode powers for the Sc and Al targets were set at 700 and 900 W during deposition, with a nitrogen flow rate of 32 sccm.

For the patterning of top and bottom Al poling electrodes, Microposit®s1813® photoresist was used. The photoresist was spin-coated at 3000 rpm and baked at 115 °C for 1 min before exposing to a contact lithography tool. The resist was developed using microposit MF CD-26 developer at room temperature. To protect the thin film Al from the possible attack of photoresist developer (MF CD-26 developer), a layer of polymer (ZEP 520 A) was spin-coated at 2500 rpm and baked at 120 °C before coating and developing the photoresist. After the photoresist development, the polymer (ZEP 520A) was etched using O_2_ plasma to expose the thin film Al. Both the masking layers (polymer and photoresist) after patterning of Al were cleaned using microposit remover 1165 at 65 °C.

### De-embedding technique used to obtain the resonator's intrinsic response from the measured data

It should be noted that the data extracted using the VNA contains the interconnects to the GS probe in addition to the intrinsic resonator response. De-embedding was performed to obtain the reflection factor of the intrinsic resonator

6 $${\Gamma }_{{{{DUT}}}}=\frac{A-{\Gamma }_{{{M}}}}{{A}^{2}-A{\Gamma }_{{{M}}}-B}$$

from the measured reflection factor (${\Gamma }_{{{M}}}$) using the parameters

7 $$A=\frac{{\Gamma }_{{{M,{open}}}}+{\Gamma }_{{{M,{short}}}}}{2+{\Gamma }_{{{M,{open}}}}-{\Gamma }_{{{M,{short}}}}}$$

and

8 $$B=({\Gamma }_{{{M,{open}}}}-A)(1-A)$$

that were extracted using equivalent open (Γ_M,open_) and short (Γ_M,short_) structures produced on the same Si-wafer^28^.

## Supplementary information

Supplemental Material
