# Supplementary material for: Periodically poled aluminum scandium nitride bulk acoustic wave resonators and filters for communications in the 6G era
Source: Microsyst Nanoeng. 2025 Jan 22;11:19. doi: 10.1038/s41378-024-00857-4 (PMC11754792; doi:10.1038/s41378-024-00857-4)
Supplement: Supplementary file 1 — Supplemental Material [file 41378_2024_857_MOESM1_ESM.docx]

**Supplementary Data**

# Periodically Poled Aluminum Scandium Nitride Bulk Acoustic Wave Resonators and Filters for Communications in the 6G Era

*Izhar^1^, M. M. A. Fiagbenu^1^, S. Yao^1^, X. Du^1^, P. Musavigharavi^1,2^, Y. Deng^1^, J. Leathersich^3^, C. Moe^3^, A. Kochhar^3^, E. A. Stach^4^, R. Vetury^3^, and R. H. Olsson III^1,^*

^1^Department of Electrical and Systems Engineering, University of Pennsylvania, Philadelphia, PA 19104 USA

^2^Department of Materials Science and Engineering, University of Central Florida, Orlando, FL 32816 USA

^3^Akoustis Inc., Huntersville, NC 28078 USA

^4^Department of Materials Science and Engineering, University of Pennsylvania, Philadelphia, PA 19104 USA

**Corresponding authors:**

Prof. Roy H. Olsson III

Email: [rolsson@seas.upenn.edu](mailto:rolsson@seas.upenn.edu)

Office: +1(215) 898-6424

The current vs. voltage response recorded during the electrical poling of the AlScN (layer 3) in fabrication step IV, depicted in Fig. S1a, showing the transition of AlScN (layer 3) from N-polar to M-polar at a coercive field voltage of 150 V. Due to increased capacitance of the devices with number of the BAW resonators, the current observed for 3 and 6-element filters is larger compared to single BAW resonator.

Also, as shown in Fig. S1b, the current vs. voltage response recorded during electrical poling shows that polarity switching is reversible (the device can be poled from the M-polar to the N-polar state and from the N-polar to the M-polar state). Further, the triangular voltage and current waveforms, as shown in Fig. S1c, shows that the device poled into the N-polar state at point 1 during the first triangular pulse. The absence of this current in the second triangular pulse confirms the AlScN is completely poled. The switching current at point 3 confirms that device is poled into the M-polar state during the application of third triangular pulse. The absence of this current in the fourth triangular pulse confirms that the AlScN is completely poled and stays in M-polar state.

**a**

Fig. S1| **a** the current vs. voltage response recorded during the electrical poling of the AlScN (layer 3) showing the transition of AlScN (layer 3) from N-polar to M-polar at a coercive field voltage of 150 V, **b** the current vs. voltage response recorded during electrical poling showing that polarity switching is reversible, **c** the triangular voltage and current waveforms showing (1) device poled into N-polar state, (2) lack of switching current confirms the N-polar state, (3) device poled into M-polar state, and (4) lack of switching current confirms the M-polar state.

Dimensions of the fabricated layer stack of the BAW devices are listed in Table S1.

Table S1 | Dimensions of the fabricated BAW devices.

| Parameters | Values | |
| --- | --- | --- |
|  | Series | Shunt |
| Si_3_N_4_ | 20 nm | 20 nm |
| Mo | ~100 nm | ~100 nm |
| AlScN Epi | 124 nm | 124 nm |
| AlScN | 230 nm | 230 nm |
| Al | 40 nm | 40 nm |
| AlScN | 223 nm | 223 nm |
| AlScN | 115 nm | 115 nm |
| Mo | ~100 nm | ~100+20 nm |
| SiO_2_ | 50 nm | 50 nm |

The phase angle of both the series and shunt resonators is also in close agreement with the mBVD model data as depicted in supplementary Fig. S2a and Fig. S2b, respectively.

**a b**

Fig. S2| Phase angle of **a** series and **b** shunt resonators.

The Smith chart of the series and shunt resonators are reported in Fig. S3a and Fig. S3b, respectively which shows close agreement between experimental results and the mBVD model.

**a b**


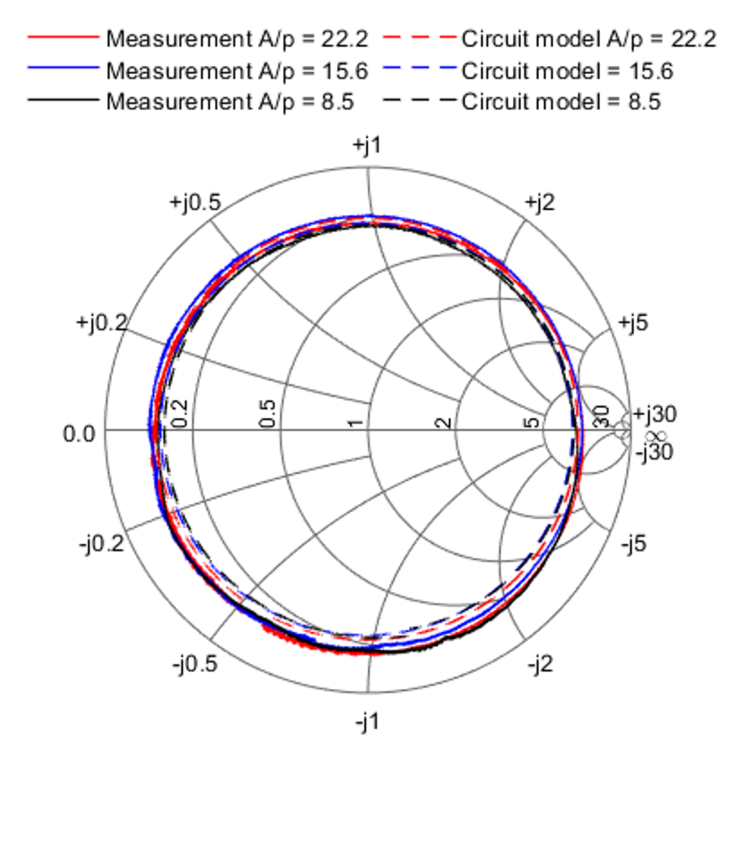

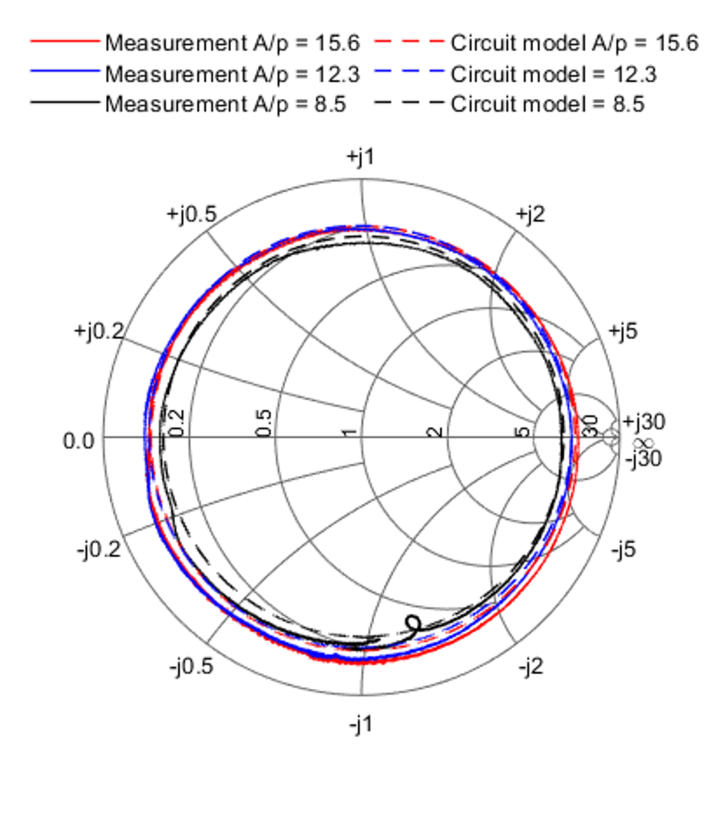


Fig. S3| Smith charts of **a** series and **b** shunt resonators.

The bode Q of the series and shunt resonators are reported in Fig. S4a and Fig. S4b, respectively which shows close agreement between experimental results and the mBVD model.

**a b**

Fig. S4| Bode Q of **a** series and **b** shunt resonators.

The stress distributions of the resonator material stack simulated in COMSOL Multiphysics at ~ 3.5 GHz, 8 GHz and 12 GHz frequencies, as shown in Fig. S5a-c, confirm the TE1, TE2 and TE3 modes of operation of the device, respectively. Note that the stress distribution of the acoustic standing waves at these frequencies do not match the periodically poled pattern of the AlScN P3F, resulting in reduced k_t_^2^ for these modes.

Fig. S5| COMSOL Simulations. Stress distributions of the acoustic standing waves in the resonators at **a** 3.5 GHz, **b** 8 GHz, and **c** 12 GHz frequencies respectively corresponding to the TE1, TE2 and TE3 modes of operation.

The IIP3 measurements of the filters were performed using the experimental setup shown in Fig. S6c. The two-tone signals ($f_{1}$and $f_{2}$) were produced in the testing setup using two signal generators (Keysight P5026B) for IIP3 measurement.^33^ These signals were then amplified using a Minicircuit ZVM-273HP+ for in-band IIP3 measurement. Two filters (ZLSS-K24G+) and two circulators (D3C8020) were utilized to remove amplifier intermodulation products (filters) and to attenuate reflections (circulators) before and after the amplifiers. A power combiner (Krytar 6020265) was used to combine these two signals, followed by another circulator, and finally to the filters. The input power at the filter was calibrated using a thru structure. The output of the filter device was connected to another circulator and then to the spectrum analyzer (Keysight P5026B) for measurement.

Fig. S6| Experimental setups. Testing setup for characterization of **a** P3F AlScN resonators and **b** filters showing the devices are connected to the VNA using GS probes for data collection. **c** schematic of the IIP3 measurement setup.

The response of a standard thru structure on a calibration substrate showing that our IIP3 measurement is limited by the experimental setup, as illustrated in Fig. S7.

Fig. S7| The response of a standard thru structure on a calibration substrate showing that our IIP3 measurement is limited by the experimental setup.

The response of 3-element filters at different input power levels is shown in Fig. S6. The filter shows linear operation up to +20 dBm, which is the limit of our test equipment.


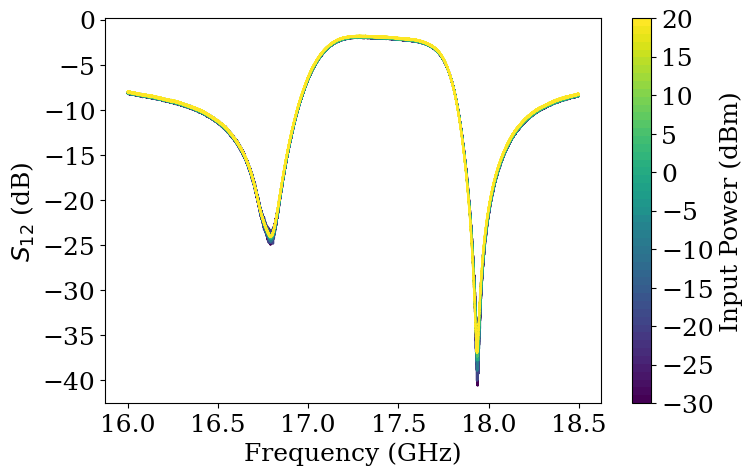


Fig. S8| Response of 3-element filter at different input power levels showing linear operation up to +20 dBm, which is the limit of our test equipment.

The response of the P3F resonator compared to an identical unpoled resonator as shown Fig. S9. It is clear from the figure, that the unpoled device shows a dominant response at the TE1 mode, whereas the electrically poled P3F device shows a dominant response at the TE4 mode as designed, which confirms the successful polarity of the P3F devices.

Fig. S9| Response of identical unpoled and electrically poled P3F resonators showing dominant responses at the TE1 mode and TE4 modes, respectively.
